# Organ dysfunction as a new standard for defining sepsis

**DOI:** 10.1186/s41232-016-0029-y

**Published:** 2016-11-15

**Authors:** Seitaro Fujishima

**Affiliations:** Center for General Medicine Education, Keio University School of Medicine, Tokyo, Japan

**Keywords:** Sepsis-3, Sequential organ failure assessment score, Acute respiratory distress syndrome, Acute kidney injury, Disseminated intravascular coagulation

## Abstract

Despite advances in intensive care and the widespread use of standardized care included in the Surviving Sepsis Campaign Guidelines, sepsis remains a leading cause of death, and the prevalence of sepsis increases concurrent with the aging process. The diagnosis of sepsis was originally based on the evidence of persistent bacteremia (septicemia) but was modified in 1992 to incorporate systemic inflammatory response syndrome (SIRS). Since then, SIRS has become the gold standard for the diagnosis of sepsis. In 2016, the Society of Critical Care Medicine and the European Society of Intensive Care Medicine published a new clinical definition of sepsis that is called Sepsis-3. In contrast to previous definitions, Sepsis-3 is based on organ dysfunctions and uses a sequential organ failure (SOFA) score as an index. Thus, patients diagnosed with respect to Sepsis-3 will inevitably represent a different population than those previously diagnosed. We assume that this drastic change in clinical definition will affect not only clinical practice but also the viewpoint and focus of basic research. This review intends to summarize the pathophysiology of sepsis and organ dysfunction and discusses potential directions for future research.

## Background

There have been significant advances in intensive care and the widespread use of standardized care in the Surviving Sepsis Campaign Guidelines. However, sepsis remains a leading cause of death and its prevalence increases concurrent with the aging process. In 2016, the Society of Critical Care Medicine and the European Society of Intensive Care Medicine published a new clinical definition of sepsis that is termed as Sepsis-3. This new definition was subsequently endorsed by a range of affiliated societies, including the Japanese Society of Intensive Care Medicine and the Japanese Association for Acute Medicine (JAAM). The consensus of opinion indicates that Sepsis-3 will lead to significant changes in the concept of sepsis [1].

**Fig. 1:**
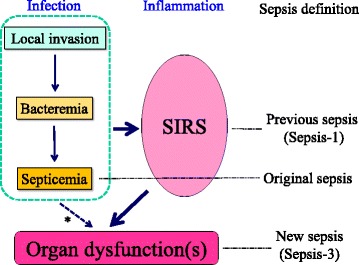
Schematic diagram showing the previous and new definitions of sepsis. *Asterisk* indicates a small fraction of infected patients develop organ dysfunction without fulfilling the established SIRS criteria. *SIRS* systemic inflammatory response syndrome

The diagnosis of sepsis was historically based on the evidence of persistent bacteremia (septicemia). However, in accordance with the progress in our understanding of the pathophysiology of sepsis, the definition was greatly modified in 1992 to exclude bacteremia and to incorporate parameters related to systemic inflammation (Sepsis-1), namely the systemic inflammatory response syndrome (SIRS) [2] (Fig. 1). At the same time, two severe subgroups of sepsis were defined as severe sepsis and septic shock. Although the definition of sepsis was reevaluated and modified to include a wider range of parameters in 2003 (Sepsis-2), SIRS continues to be widely used for diagnosing sepsis in various clinical settings [3]. However, the inadequate specificity and sensitivity of the SIRS criteria were recognized as a significant limitation and, thus, were omitted from Sepsis-3 [4]. In contrast to previous definitions, Sepsis-3 is based on organ dysfunctions and sepsis is diagnosed when there is an increase of more than 2 points in the total sequential organ failure assessment (SOFA) score; thus, patients diagnosed with respect to Sepsis-3 will inevitably represent a different population from those diagnosed with respect to Sepsis-1 or Sepsis-2. Therefore, we assumed that this drastic change in clinical definition will not only affect clinical practice, especially the evaluation of patients with suspected infection, but also lead to changes in the focus of basic research. In this review, we intend to provide an overview of the pathophysiology of sepsis-induced organ dysfunction and elucidate the present unresolved questions for indicating the direction of the research in this field.

## Pathophysiology of sepsis

Infection is initiated by the local invasion of microorganisms into a living body. When the host’s immune system is healthy and the quantity and virulence of the microorganisms involved are below a tolerance limit, infection is restricted and spontaneously cures. However, if microorganisms overcome a host’s immunological self-defense system, they can locally extend or spread to distant tissues and organs via the blood stream. In response to the systemic invasion of microorganisms, the body triggers the production of inflammatory mediators and characteristic signs and symptoms, such as fever or hypothermia, tachycardia, tachypnea, and an increase or decrease in peripheral white blood cell counts develop, all of which are essential components of the SIRS criteria. It was at this stage that the diagnosis of sepsis was made with respect to the former definition, which often predisposed the failure of vital organs, currently referred to as multiple organ dysfunction syndrome (MODS). Thus, sepsis diagnosed with respect to the Sepsis-3 definition, namely infection-induced single or multiple organ dysfunctions, is more advanced and manifests with more severe conditions than that diagnosed by previous definitions.

Pathogen-derived substances with immunological properties, notably lipopolysaccharide (LPS), are referred to as pathogen-associated molecular patterns (PAMPs) and have been shown to induce sepsis-like conditions in vivo and activate immune cells in vitro [5]. Injured tissues also release a set of molecules with similar properties, including high-mobility group box 1 (HMGB-1) and histones, collectively referred to as damage (danger)-associated molecular patterns (DAMPs) [6]. Under septic conditions, PAMPs and DAMPs bind to specific cell-surface or cytosolic proteins that are called pattern recognition receptors, which are located on monocytes/macrophages, vascular endothelial cells, and other stromal cells, and that trigger the activation of intracellular signaling cascades [7]. Activated cells release various humoral mediators, particularly cytokines, which accelerate local and systemic inflammations.

Cytokines are classified as pro-inflammatory and anti-inflammatory, and the time course of their expressions vary. Pro-inflammatory cytokines play major roles in inducing systemic inflammation and in the development of MODS. Among the pro-inflammatory cytokines, tumor necrosis factor alpha (TNFα) and interleukin 1beta (IL-1β) are detected in the blood of patients with sepsis and are known to induce septic shock-like conditions when administered to animals in vivo, thus suggesting their key pathogenic roles in sepsis [8, 9]. In contrast, anti-inflammatory cytokines contribute to the regulation and resolution of acute inflammation, while also contributing to the immunosuppression and hypersensitivity to infection that is observed during the later phase of sepsis.

## Sepsis-associated lung dysfunction: ARDS

Lung dysfunction, referred to as acute respiratory distress syndrome (ARDS) or acute lung injury (ALI), is frequently associated with sepsis. In our recent epidemiological analysis, ALI was complicated in 40.2 % of patients with severe sepsis or septic shock and had a significantly poor outcome [10]. A previous report revealed that sepsis-related ARDS was associated with a poorer outcome than non-sepsis-related ARDS [11]. An increase in microvascular permeability, resulting from the dysregulation of cell-to-cell interaction or tissue destruction, is the fundamental pathophysiology underlying ARDS. Extensive investigation has revealed the prominent contribution of neutrophils, which are major terminal effector cells in innate immunity, to tissue injury in ARDS [12, 13]. Neutrophils have also been shown to play a role in the dysfunction of other organs. Neutrophils release granular enzymes, reactive oxygen metabolites, bioactive lipids, and cytokines [14] and can induce the formation of neutrophil extracellular traps [15], most of which can either directly or indirectly injure tissues, leading to an increase in microvascular permeability and resulting in pulmonary edema. Interleukin 8 (also called CXCL8), a potent neutrophil chemotactic chemokine, also plays important roles in the pathophysiology of ARDS, along with TNFα and IL-1β, which represent major players in septic shock [16]. In addition, some DAMPs have been recently recognized as mediators or cytokines with respect to their regulated production and immune-regulating functions. A pathogenic role of HMGB-1, a molecule originally identified as a nuclear binding protein, was initially reported in sepsis [17] and later in sepsis-associated ARDS [18]. Mitochondrial DAMPs can stimulate PMNs and induce a sepsis-like state and ALI [19]. Along with neutrophil-mediated tissue injury, apoptosis and autophagy are also involved in sepsis-induced tissue injuries that are associated with ARDS [20, 21]. However, these findings did not lead to the development of appropriate clinical therapeutics.

## Dysfunction of other organs in sepsis

As mentioned in the above section, sepsis is often complicated with organ dysfunctions other than ARDS. Table 1 summarizes the pathophysiology, clinical features, SOFA score indices, and available treatments for individual organ dysfunctions. The type of failure or dysfunction observed in each organ is relatively stereotyped and most are associated with poor outcomes [22, 23]. Sepsis-derived systemic shock and resultant tissue hypoperfusion commonly contribute to the development of most organ dysfunctions, which is supported by the findings that early goal-directed therapy (EGDT), a protocol-based therapeutic intervention involving fluid resuscitation, improved the MODS score and outcome [24]. Furthermore, plasma pro-inflammatory cytokine levels were significantly reduced in an EGDT group compared with a control group, suggesting that global tissue ischemia is an important contributor to the early inflammatory response in sepsis [25]. However, as the mere recovery of systemic circulation by fluid resuscitation and inotropic agents is insufficient to completely restore organ function, other injurious mechanisms are most certainly involved.

**Table 1:** Organ dysfunction in sepsis

Target organ	Pathophysiology	Clinical features	SOFA score indices (other beneficial indices)	Available treatments
Lung (ARDS)	Vascular hyper-permeability, neutrophil accumulation	Impaired oxygenation	PaO_2_/FIO_2_ <400 (bilateral infiltration on CXR)	Mechanical ventilation with low tidal volume and PEEP
Liver	Disturbed intracellular and extracellular bile salt transport	Jaundice, cholestasis	Serum bilirubin ≥1.2 mg/dl	Not established
Kidney (AKI)	Tubular epithelial cell injury, dysfunction or adaptive response of tubular epithelial cells	Reduced GFR, reduced urine volume	Serum creatinine ≥1.2 Urine output <500 ml/day	Hemodialysis
Cardiovascular system	Myocardial depression, impaired intracellular calcium homeostasis, disrupted high energy phosphate production.	Ventricular dilatation, reduced ejection fraction, reduced contractility	Mean arterial pressure <70 mmHg	Inotropic agents, beta-blocker
Gastrointestinal tract	Epithelial hyper-permeability, altered microbiome	Mucosal bleeding, paralytic ileus	Not included	Proton pump inhibitor, early enteral nutrition, probiotics, SDD
Central nervous system (SAE)	Direct cellular damage, mitochondrial and endothelial dysfunction, neurotransmission disturbances, calcium dyshomeostasis	Altered mental status	GCS <15	Light sedation, early rehabilitation
Blood coagulation system (DIC)	Intravascular coagulation, microvascular damage, systemic thrombin generation, endothelial injury	Bleeding diathesis, microthrombi and tissue ischemia	Platelets <150 × 10^3^/μl (prolonged prothrombin time, increased FDP)	Antithrombin, recombinant thrombomodulin, concentrated platelet preparation

*SOFA* sequential organ failure assessment, *ARDS* acute respiratory distress syndrome, *CXR* chest X-ray, *PEEP* positive end-expiratory pressure, *AKI* acute kidney injury, *GFR* glomerular filtration ratio, *SDD* selective digestive decontamination, *SAE* sepsis-associated encephalopathy, *GCS* Glasgow coma scale, *DIC* disseminated intravascular coagulation, *FDP* fibrin degradation product

During infection, the liver plays critical roles in modulating host defense and regulating inflammation, mostly via the clearance of pathogens and PAMPs, and in producing acute phase proteins such as C-reactive protein and serum amyloid A [26, 27]. A previous study revealed through a multivariate analysis that liver cirrhosis was associated with a 2.4-fold increase in sepsis mortality [28]. Our analysis and that of other groups showed that 10–17 % of patients with severe sepsis were complicated with hepatic dysfunction [22, 23], which was shown to be associated with a poorer clinical outcome in a large observational study [23]. Sepsis-associated hepatic dysfunction is clinically recognized by jaundice or cholestasis and is caused by various underlying mechanisms such as the impairment of energy-dependent bile and bile acid transport by hypoxia, hypoperfusion, and overproduced cytokines. No treatments are currently established for such conditions.

The kidneys are the main controllers of water and electrolyte metabolism under normal physiological conditions, and the roles of the kidneys become even more important during sepsis for maintaining vital organ circulation and cellular electrolyte balance and for protecting the lungs from life-threatening pulmonary edema. Acute dysfunction of the kidneys is referred to as acute kidney injury (AKI) and was observed in 37–40 % of cases with severe sepsis [22, 23]. AKI is clinically detected as an increase in serum creatinine level or a decrease in urine volume and is associated with poorer clinical outcomes [22, 23, 28]. Renal tubular epithelial cell injury, which is because of disturbed microcirculation and hypoxia, as well as the dysfunction or adaptive response of tubular epithelial cells, including the downregulation of metabolism and cell cycle arrest, is induced by excessive inflammation and represents a key feature of AKI [29]. Although hemodialysis has been established as a clinical therapy for AKI, no strategy to prevent AKI or to help patients recover from AKI has been developed.

Blood coagulation and fibrinolysis systems contribute in the maintenance of systemic and organ circulation against various injuries to the living body. Under the condition of sepsis, these systems tend to dysfunction, a condition referred to as disseminated intravascular coagulation (DIC), which induces organ dysfunction and is closely associated with higher mortality. The major pathophysiology of DIC includes the generalized activation of intravascular coagulation, microvascular damage, systemic thrombin generation, and endothelial injury [30]. There are several diagnostic criteria for DIC, such as those by JAAM, the International Society on Thrombosis and Haemostasis (ISTH), and the Japanese Ministry of Health, Labour and Welfare, with increasingly reduced levels of sensitivity in this order. The SOFA score used in the new Sepsis-3 definition includes platelet counts as a parameter for DIC. In retrospective and prospective studies, DIC was observed in 18–41 % by ISTH and 47 % by the JAAM criteria [31–33]. Although several potentially effective drugs have been clinically used, such as antithrombin III and recombinant thrombomodulin, their efficacy in terms of survival and protection against MODS remains to be clarified.

In addition to the abovementioned organs, it is known that the functions of the cardiovascular system, gastrointestinal tract, and central nervous system are often impaired during sepsis and are crucial for patient outcome. Although some therapies, including beta-blockers for the cardiovascular system, early enteral nutrition for the gastrointestinal tract, and light sedation/early rehabilitation for the central nervous system, are potentially effective, their efficacy is limited. Consequently, new organ-specific strategies based on a novel insight into the pathophysiology should be explored.

## Future directions for sepsis research

Previous research in both animal models and patients revealed that sepsis is inevitably associated with excessive inflammation. However, anti-inflammatory strategies have failed to demonstrate clear benefit in terms of patient outcome. To begin a new era of clinical practice for sepsis, it is important to consider the appropriate direction for future research. The introduction of new clinical definition may provide us a favorable opportunity to change our emphasis with regard to research. Although the true causal relationship between individual organ dysfunction and clinical outcome remains unresolved, the extent of organ dysfunction and the total number of dysfunctional organs involved are both associated with increased mortality, rendering attempts to protect or recover from organ dysfunction as a promising approach for sepsis research. Although the lungs will continue to be the main target, all vital organs could be possible targets for research.

In most preclinical experiments on sepsis, LPS or pathogens were administered to small animals, and the post-mortem characteristics of single organs and survival rates were evaluated. However, the function of multiple organs would usually be impaired in such models and additively or synergistically affect the outcome, in a manner similar to real human patients. Consequently, in future research, simultaneous evaluation of dysfunction in more than two vital organs will be mandatory in such sepsis models. Additionally, the pathophysiology and therapeutic strategy for individual organ dysfunction should be more extensively examined using existing or newly developed single organ dysfunction models, including the septic ARDS model via the intratracheal administration of pathogens or PAMPs.

For such investigations, it is important to develop novel techniques to sequentially monitor organ function hopefully in vivo. To begin with, such research would be significantly enhanced by developing small-scale apparatus to monitor the partial pressure or saturation of arterial blood oxygen and arterial blood pressure. In addition, it would be important to incorporate measurements of serum bilirubin, creatinine, and urea nitrogen for in vivo monitoring across a range of animal models. In contrast, novel strategies need to be developed to enable the evaluation of species-specific parameters such as blood cells and proteins involved in the coagulation and fibrinolysis systems.

## Conclusions

Excessive inflammation has been established as the key feature of sepsis pathophysiology and therefore a prime target of intervention. This understanding led us to the concept of SIRS, and sepsis was defined as the infection-induced SIRS. However, anti-inflammatory strategies have failed to improve clinical outcome in patients with sepsis. As described earlier, the definition of sepsis was drastically modified in 2016 so as not to include SIRS criteria and inflammatory variables. Instead, sepsis is now clinically defined by an increase in SOFA score. Thus, patients diagnosed with Sepsis-3 will inevitably represent a population different from that previously diagnosed. We assume that this drastic change in clinical definition will affect not only clinical practice but also the direction of future basic research.

Simultaneously evaluating multiple organ dysfunction in animal models of sepsis or investigating single organ dysfunction models will enable the development of potential organ-protective treatments in a robust experimental manner. Such organ-targeted investigations will certainly strengthen our understanding of the pathophysiology of sepsis and may help to develop truly effective therapies to protect or recover from organ dysfunction in sepsis.

## Abbreviations

AKI: Acute kidney injury
ALI: Acute lung injury
ARDS: Acute respiratory distress syndrome
DAMPs: Damage (danger)-associated molecular patterns
DIC: Disseminated intravascular coagulation
HMGB-1: High-mobility group box 1
IL-1β: Interleukin 1beta
ISTH: International Society on Thrombosis and Haemostasis
JAAM: Japanese Association for Acute Medicine
LPS: Lipopolysaccharide
MODS: Multiple organ dysfunction syndrome
PAMPs: Pathogen-associated molecular patterns
SIRS: Systemic inflammatory response syndrome
SOFA: Sequential organ failure assessment
TNFα: Tumor necrosis factor alpha
